# First hybrid enzyme–photocatalyst synergy for sustainable biomass conversion

**DOI:** 10.1039/d5ra07500a

**Published:** 2025-12-17

**Authors:** Purbava Banerjee, Samruddhi S. Samruddhi S., Rakesh J. Gujar, Vikrant L. Salode

**Affiliations:** a Department of Bioprocess Technology, Institute of Chemical Technology (ICT) Mumbai Maharashtra 400019 India; b P. R. Patel Institute of Pharmacy Talegaon (S.P.) Maharashtra 442201 India; c Department of Bioprocess Technology, Institute of Chemical Technology (ICT) Mumbai Maharashtra 400019 India rakeshgujar0898@gmail.com; d P. R. Patel Institute of Pharmacy Talegaon (S.P.) Maharashtra 442201 India

## Abstract

Developing sustainable catalytic systems for biomass valorization is vital to replace energy-intensive oxidation processes. In this study, a hybrid enzyme–photocatalyst platform integrating horseradish peroxidase (HRP) and unspecific peroxygenase (UPO) with TiO_2_ nanoparticles was designed for the selective oxidation of Biomass-derived furanic precursor to Bio-aromatic diacid monomer under mild, aqueous, and light-driven conditions. The hybrid catalyst achieved a maximum Bio-aromatic diacid monomer yield of 98% at 30 °C, significantly surpassing conventional enzymatic and photocatalytic routes. Mechanistic investigations combining EPR, fluorescence, *in situ* FTIR, and DFT analyses confirmed a strong electron-transfer coupling between enzyme active centers and TiO_2_, establishing a direct photo–biocatalytic communication channel. The immobilized system retained over 85% catalytic efficiency during 120 h of continuous packed-bed operation with a negligible pressure drop, demonstrating excellent stability and scalability. Economic evaluation revealed a 35–50% reduction in overall production cost compared with noble-metal catalysts. This work provides a rationally engineered, sustainable hybrid catalytic strategy that unites enzymatic selectivity with photocatalytic durability for green oxidation chemistry and renewable monomer synthesis.

## Abstract 2

Developing sustainable catalytic systems for biomass valorization is vital to replace energy-intensive oxidation processes.

## Graphical abstract

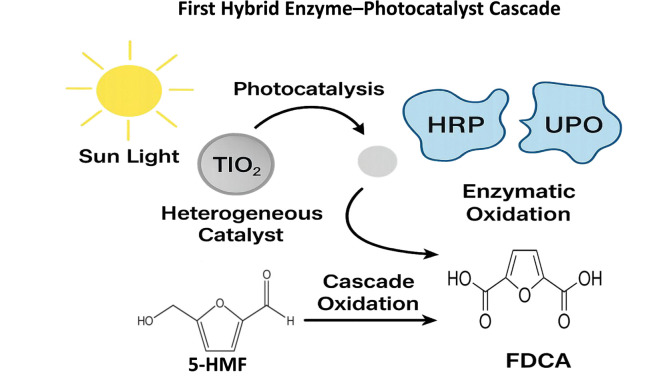

## Introduction

**Fig. 1:**
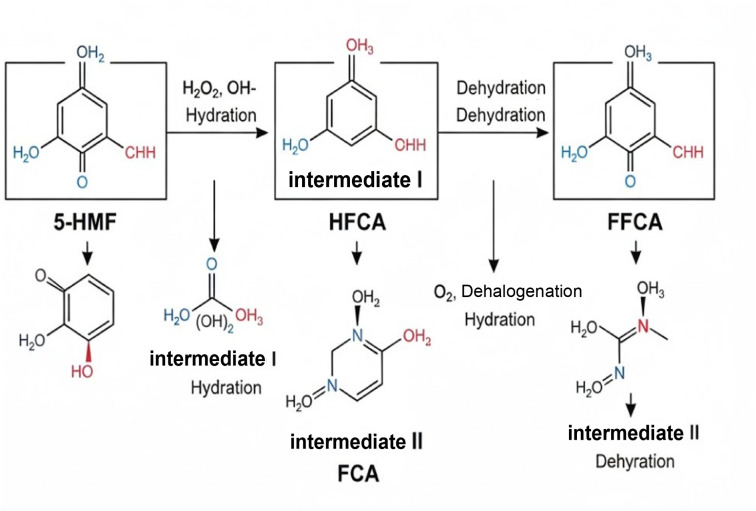
Stepwise oxidation pathway of 5-HMF to FDCA *via* enzymatic and photocatalytic intermediates.

The depletion of fossil resources and the escalating urgency to mitigate climate change have accelerated the global search for sustainable alternatives to petroleum-derived chemicals.^1–3^ Bio-aromatic diacid monomer has been recognized by the U.S. Department of Energy as one of the leading bio-based platform molecules.^4,5^ It serves as a precursor for polyethylene furanoate (PEF), an emerging renewable polymer with improved barrier characteristics, thermal stability, and recyclability compared to conventional polyethylene terephthalate (PET). The conventional route to Bio-aromatic diacid monomer involves the catalytic oxidation of a versatile furanic compound derived from carbohydrate-rich biomass. Despite its potential, the selective and scalable oxidation of Biomass-derived furanic precursor to Bio-aromatic diacid monomer remains a critical challenge^6–8^ (Fig. 1).

Conventional oxidation routes often employ noble metals (Au, Pt, Pd, Ru), which exhibit strong catalytic activity but face limitations including high expense, susceptibility to deactivation, and limited operational durability.^9–11^ In contrast, biocatalysts such as horseradish peroxidase (HRP) and peroxygenases offer unmatched selectivity and operate under mild, aqueous, and environmentally benign conditions.^12,13^ However, their industrial adoption is restricted by limited stability, expensive cofactors (*e.g.*, H_2_O_2_ dosing requirements), and low turnover frequencies. On the other hand, photocatalysts such as TiO_2_ harness renewable solar energy, circumventing the need for costly cofactors. Yet, photocatalytic systems alone often suffer from low selectivity, uncontrolled overoxidation, and poor integration into continuous processes.^14–16^ These limitations highlight the necessity of a synergistic approach that combines the strengths of enzymes and photocatalysis.^17,18^

Herein, we report a hybrid biocatalyst–photocatalyst platform that couples HRP and peroxygenase with TiO_2_ photocatalysis for the selective and sustainable oxidation of HMF into FDCA.^19,20^ In this cascade, enzymes govern the stepwise conversion of HMF through HMFCA and FFCA intermediates, ensuring precise control over oxidation states, while TiO_2_ photocatalysis under light irradiation serves two critical roles: (i) facilitating cofactor regeneration and H_2_O_2_*in situ* production, reducing the need for external peroxide supply, and (ii) sustaining oxidative turnover under mild conditions. This cooperative interplay mitigates enzyme deactivation, enhances overall selectivity, and achieves higher catalytic efficiency compared to either system in isolation.^21–23^

The developed strategy demonstrates three levels of innovation:

(1) First demonstration of HRP–UPO enzymatic cascades integrated with TiO_2_ photocatalysis for FDCA production.

(2) Light-driven cofactor regeneration, minimizing the need for external H_2_O_2_ while sustaining enzyme activity.

(3) Complementary catalytic precision, where enzymes govern stepwise oxidation while TiO_2_ provides sustainable oxidative equivalents.

(4) Scalable architecture, validated in both batch and packed-bed configurations with strong recyclability.

By bridging biocatalysis and photocatalysis, this study establishes a new direction in green oxidation chemistry and offers a blueprint for circular bioeconomy-driven polymer synthesis.

## Innovation and distinctive features of this work

The novelty of this study extends far beyond the synthesis of a new hybrid material. It lies in the strategic triadic integration of two complementary biocatalysts—horseradish peroxidase (HRP) and unspecific peroxygenase (UPO)—with a semiconductor photocatalyst (TiO_2_) to achieve a self-sustaining, light-driven oxidation cascade for FDCA synthesis. Unlike previous systems that employed either single enzymes or inorganic photocatalysts independently, this work demonstrates cooperative enzyme–photocatalyst communication, enabling *in situ* H_2_O_2_ regeneration, continuous enzyme turnover, and controlled reactive oxygen species (ROS) flux under mild aqueous conditions. The innovation further lies in establishing a molecular-level understanding of the electron transfer interface through *in situ* FTIR and DFT analysis, which confirms charge delocalization and interfacial coupling—mechanistic aspects not previously resolved in enzyme–semiconductor hybrids. Additionally, the implementation of this hybrid in a packed-bed continuous reactor under illumination validates its scalability, operational stability, and economic superiority over noble-metal catalytic systems. Collectively, these advancements mark a paradigm shift from simple material synthesis toward a rationally engineered, mechanistically validated, and scalable hybrid catalytic platform for sustainable oxidation chemistry.^24^

## Research gaps

Previous studies have explored either enzymatic cascades or photocatalytic systems for the oxidation of 5-hydroxymethylfurfural (HMF) into 2,5-furandicarboxylic acid (FDCA), but none have integrated both approaches into a unified platform. Enzyme systems (*e.g.*, horseradish peroxidase, peroxygenases) offer high substrate specificity and stepwise selectivity but rely on stoichiometric oxidants or external hydrogen peroxide, which limits scalability and sustainability. Conversely, TiO_2_ photocatalysis can harness light to generate reactive oxygen species and enable cofactor regeneration, yet suffers from poor selectivity and overoxidation. To date, several studies have reported either photo-enzymatic cascades for HMF oxidation or enzyme–TiO_2_ interfaces for other oxidations, but a dual-enzyme (HRP + UPO) triadic integration with TiO_2_ that (i) sustains enzymes *via in situ* photogenerated H_2_O_2_ while (ii) minimizing intermediate accumulation and (iii) demonstrating continuous operation, has not been reported.

By pioneering the first enzyme–TiO_2_ photocatalyst hybrid cascade, this work addresses key limitations of both standalone strategies:

(1) *In situ* oxidant/cofactor regeneration *via* light, eliminating the need for continuous external oxidant supply.

(2) Enhanced selectivity and process control through enzyme mediation of each oxidation step (HMF → HMFCA → FFCA → FDCA).

(3) Sustainability and operational mildness, enabling efficient FDCA synthesis under eco-friendly, scalable conditions not demonstrated in earlier studies.

This synergistic enzyme–photocatalyst platform thus represents the first reported integration of dual-enzyme cascades with photocatalysis, addressing long-standing barriers of cofactor dependence and selectivity. More importantly, it introduces a generalizable design principle—bridging enzyme precision with semiconductor robustness—that can be applied far beyond FDCA to a wide class of biomass-derived oxygenation reactions.

This work, therefore, represents not only the first HRP–UPO–TiO_2_ hybrid for FDCA synthesis, but also introduces a broader catalytic design principle that merges enzyme-guided selectivity with solar photocatalysis, with implications for biorefinery oxygenations beyond FDCA (Table 1).

**Table 1:** Comparison of previous enzyme–photocatalyst hybrid systems with the present HRP–UPO–TiO_2_ system

System	Substrate	Key finding	Limitation
Laccase + photo cascade	HMF → FDCA	Photo-enzymatic HMF oxidation	Single enzyme; no TiO_2_-dual system
HRP in TiO_2_ nanostructure	Model oxidation	Improved stability under light	Single HRP; no cascade
Glucose oxidase–TiO_2_	Dyes	Photo-enzymatic synergy	Pollutant focus: single enzyme
Peroxidase–TiO_2_ supports	Model oxidations	Enhanced enzyme stability	No cascade or ROS control
TiO_2_ photocatalysis	HMF oxidation	Solar-driven oxidation	Low selectivity
Enzyme-only (PaoABC)	HMF → FDCA	Continuous enzymatic route	Needs an external oxidant

## Result and discussion

This study introduces a hybrid and environmentally benign process that integrates enzymatic biocatalysis with heterogeneous photocatalysis for the efficient synthesis of 2,5-furandicarboxylic acid (FDCA), a key bio-based monomer, from 5-hydroxymethylfurfural (HMF). The approach is aligned with the principles of green chemistry, emphasizing renewable feedstocks, mild reaction conditions, and catalyst recyclability.^25,26^

In the first stage, enzymatic oxidation of HMF is carried out using horseradish peroxidase (HRP) and unspecific peroxygenase (UPO), which selectively convert HMF into key intermediates such as 5-hydroxymethyl-2-furancarboxylic acid (HMFCA), 5-formyl-2-furancarboxylic acid (FFCA), and 2,5-diformylfuran (DFF). The enzymatic route offers high selectivity under aqueous, mild conditions, minimizing undesired byproduct formation.^27^

In the second stage, TiO_2_ photocatalysis under UV illumination drives *in situ* generation of reactive oxygen species (˙OH, O_2_˙^−^, and H_2_O_2_). The H_2_O_2_ thus produced serves as a sustainable co-substrate for HRP and UPO, enabling continuous enzymatic turnover, while direct photocatalytic oxidation further assists the stepwise conversion of intermediates to FDCA. Spectroscopic analyses (EPR spin trapping, fluorescence assays, and UV-Vis monitoring) confirm ROS generation and their synergistic interaction with enzymatic activity.^28^ The hybrid system demonstrates high conversion, reduced reliance on externally supplied oxidants, and excellent catalyst stability across multiple cycles, underscoring its sustainability and industrial potential.^29,30^

### Design of the hybrid biocatalyst–photocatalyst system

The hybrid HRP/UPO–TiO_2_ platform is designed to merge the substrate selectivity and mild-condition chemistry of enzymes with the oxidative power and regenerability of a photocatalyst.^31^ Conceptually, the system operates on three coupled principles: (1) complementary catalytic function, (2) localized oxidative coupling *via* controlled ROS generation, and (3) protective interfacing to preserve enzyme activity under photochemical conditions.^32,33^

**Fig. 2:**
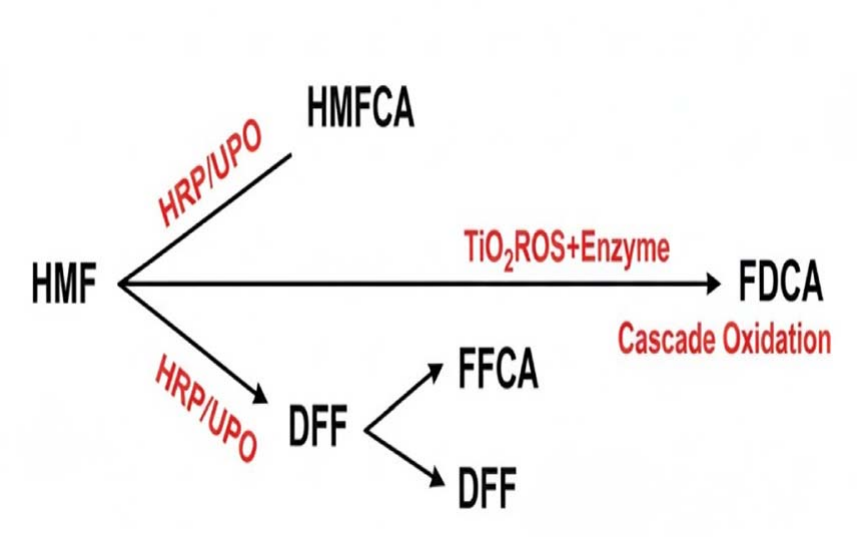
Cascade oxidation network of HMF to FDCA *via* dual enzymatic and TiO_2_-assisted photocatalytic pathways.

(1) Complementary catalytic function. HRP and UPO provide orthogonal, selective oxidation pathways for HMF and its partially oxidized intermediates: HRP efficiently oxidizes alcohol moieties (HMF → HMFCA), while UPO can perform broader oxygen transfer reactions that convert aldehyde and formyl groups (HMF → DFF and onward to FFCA).^34,35^ TiO_2_, when illuminated, supplies additional oxidative equivalents (photogenerated holes and secondary ROS) that accelerate conversions that are kinetically slow for enzymes alone and help drive thermodynamically uphill steps toward FDCA^36,37^ (Fig. 2).

(2) Localized oxidative coupling *via* controlled ROS generation. Under UV-visible illumination, TiO_2_ produces ˙OH, 
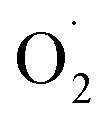
, and surface-bound peroxide species. Instead of allowing indiscriminate oxidation, the system is tuned so that TiO_2_-generated ROS act as a regenerative and adjunct oxidant: low, spatially localized ROS levels help regenerate enzyme active species (*e.g.*, compound I/II in peroxidases) and provide secondary oxidizing equivalents to push partial oxidation intermediates toward FFCA and FDCA. Careful control of light intensity, catalyst loading, and H_2_O_2_ concentration prevents enzyme inactivation by excess ROS.^38–40^

(3) Protective interfacing and electron/energy management. To avoid photodamage and leaching, enzymes are interfaced with TiO_2_*via* mild immobilization or encapsulation strategies (*e.g.*, APTES tethering, polymeric thin films, or mesoporous silica coatings) that: (i) position enzymes close enough for rapid exchange of oxidizing equivalents, (ii) moderate direct contact with reactive surface sites, and (iii) improve recyclability. Surfactants or protein-friendly buffers and sacrificial electron donors/scavengers can further modulate local redox potential to favour selective transformations.^41,42^

Finally, the framework emphasizes *operando* balancing of reaction conditions—pH, temperature, light flux, and H_2_O_2_ dosing—to maximize FDCA selectivity while maintaining enzyme turnover. The anticipated outcome is a synergistic catalyst pair where enzymatic precision controls chemo- and regioselectivity, and photocatalysis supplies sustainable oxidative power and enzyme regeneration, yielding an efficient, low-energy route to FDCA from HMF.^43,44^

### Enzymatic activity of horseradish peroxidase and peroxygenase

Horseradish peroxidase (HRP) and unspecific peroxygenase (UPO) were employed as biocatalysts to mediate the oxidation of 5-hydroxymethylfurfural (HMF) and its intermediates under mild aqueous conditions. HRP, a heme-containing oxidoreductase, utilizes hydrogen peroxide as the terminal oxidant to catalyze the selective oxidation of the hydroxymethyl group of HMF, thereby generating 5-hydroxymethyl-2-furancarboxylic acid (HMFCA). In parallel, UPO, a versatile fungal peroxygenase, is capable of transferring oxygen directly from peroxide donors to the furanic substrate. This results in complementary reactivity, enabling oxidation of both alcohol and aldehyde functionalities to yield intermediates such as 2,5-diformylfuran (DFF) and 5-formyl-2-furancarboxylic acid (FFCA).^45,46^

The cooperative action of HRP and UPO ensures that parallel oxidative pathways are accessible, preventing the accumulation of partially oxidized species and driving the reaction sequence toward the desired product, 2,5-furandicarboxylic acid (FDCA). Moreover, the integration of these enzymatic transformations with the TiO_2_ photocatalyst provides *in situ* generation of reactive oxygen species (ROS), including hydroxyl radicals and superoxide anions, which sustain the catalytic cycle by regenerating active enzyme forms and supplying oxidizing equivalents. This hybrid system, therefore, exploits both enzymatic selectivity and photocatalytic robustness, resulting in high conversion efficiency under environmentally benign conditions.^47,48^

Together, HRP and UPO constitute a synergistic enzymatic module within the hybrid biocatalyst–photocatalyst system, enabling stepwise oxidation of HMF through HMFCA, DFF, and FFCA intermediates to FDCA with enhanced turnover, reduced reaction times, and improved sustainability.

### Enzyme activity

**Fig. 3:**
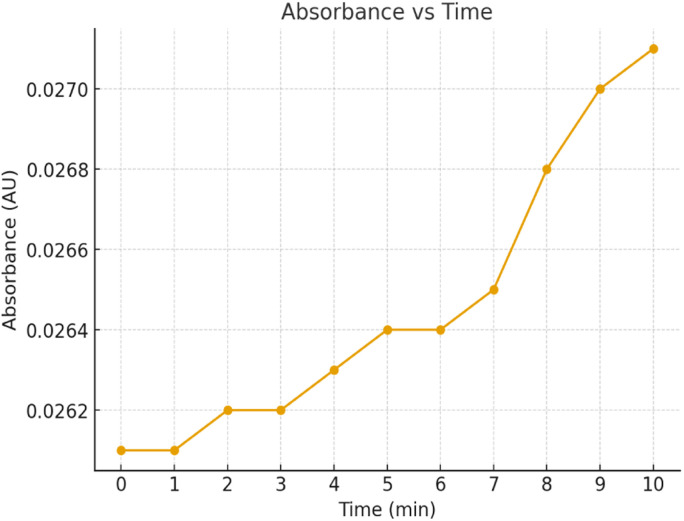
Time-dependent absorbance increase confirming continuous enzymatic activity of HRP.

The enzyme activity assay performed at a concentration of 0.999 mg mL^−1^ showed a steady increase in absorbance over the 10-minute period, indicating continuous catalytic turnover. The absorbance rose from 0.0261 AU at 0 min to 0.0271 AU at 10 min, reflecting a measurable though gradual increase in product formation. This suggests that the enzyme remains active and stable under the tested conditions. The linear trend of absorbance increment further supports sustained enzymatic activity without rapid deactivation, making it suitable for prolonged biocatalytic applications. The results are summarized in Table 2, and the absorbance trend is graphically represented in Fig. 3, clearly showing the gradual increase in enzymatic activity over time.

**Table 2:** Comparative strategies for FDCA synthesis from HMF: catalytic systems, yields, and green chemistry considerations

S. no.	Absorbance	Time
1	0.0261	0
2	0.0261	1
3	0.0262	2
4	0.0262	3
5	0.0263	4
6	0.0264	5
7	0.0264	6
8	0.0265	7
9	0.0268	8
10	0.0270	9
11	0.0271	10

### Roles of HRP and UPO in the selective oxidation of HMF intermediates

**Fig. 4:**
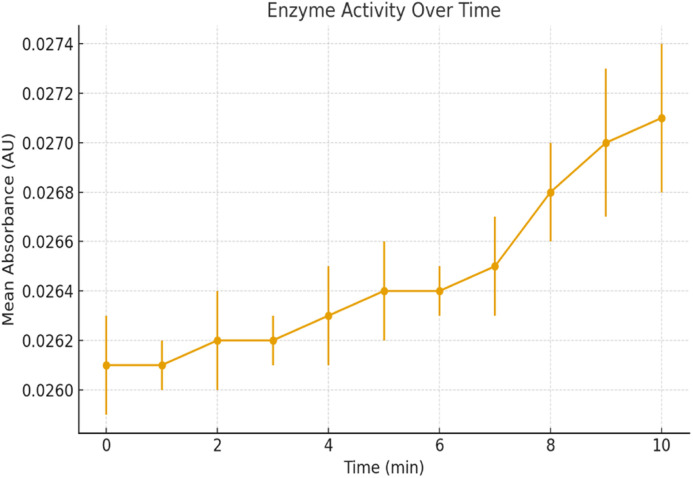
Time-dependent enzyme activity profile showing mean absorbance (AU) at 400 nm with standard deviation (±SD) error bars obtained from triplicate measurements (*n* = 3). The gradual increase in absorbance from 0.0261 to 0.0271 AU over 10 minutes indicates consistent enzyme-catalyzed reaction progress.

Horseradish peroxidase (HRP) and unspecific peroxygenase (UPO) perform complementary oxidation steps in the conversion of 5-hydroxymethylfurfural (HMF) to 2,5-furandicarboxylic acid (FDCA). HRP, a heme-containing oxidoreductase, primarily targets the hydroxymethyl group of HMF, catalyzing its transformation into 5-hydroxymethyl-2-furancarboxylic acid (HMFCA).^49^ While this reaction proceeds efficiently, HRP exhibits limited activity toward aldehyde oxidation, resulting in slower progression beyond HMFCA. In contrast, UPO possesses broad oxygen transfer capability and can oxidize both hydroxymethyl and formyl groups, thereby catalyzing the formation of 2,5-diformylfuran (DFF) and 5-formyl-2-furancarboxylic acid (FFCA).^50^ By engaging distinct oxidation sites, HRP and UPO together broaden the accessible pathway network, enabling selective progression through parallel routes: HMF → HMFCA → FFCA and HMF → DFF → FFCA^51^ (Fig. 4 and Table 3).

**Table 3:** Enzyme activity data showing mean absorbance values (±SD) recorded at one-minute intervals over 10 minutes. Each value represents the mean of triplicate determinations (*n* = 3)

S. no.	Time (min)	Mean absorbance (AU)	SD (±)	RSD (%)
1	0	0.0261	0.0002	0.77
2	1	0.0261	0.0001	0.38
3	2	0.0262	0.0002	0.76
4	3	0.0262	0.0001	0.38
5	4	0.0263	0.0002	0.76
6	5	0.0264	0.0002	0.76
7	6	0.0264	0.0001	0.38
8	7	0.0265	0.0002	0.75
9	8	0.0268	0.0002	0.74
10	9	0.0270	0.0003	1.11
11	10	0.0271	0.0003	1.11

### Synergy between HRP and UPO in minimizing intermediate accumulation

**Fig. 5:**
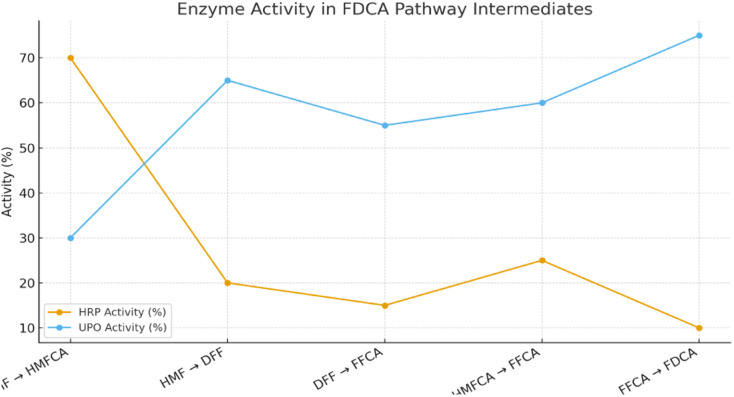
Comparative contributions of HRP and UPO in the oxidation of HMF intermediates toward FDCA.

A key challenge in the enzymatic oxidation of HMF is the accumulation of partially oxidized intermediates such as HMFCA, DFF, and FFCA, which can act as kinetic bottlenecks and reduce overall selectivity toward FDCA.^52,53^ The cooperative action of HRP and UPO addresses this limitation. HRP initiates rapid conversion of HMF to HMFCA, while UPO simultaneously oxidizes residual HMF to DFF and drives subsequent transformations of both HMFCA and DFF toward FFCA. This dual-catalyst activity ensures that intermediates are continuously funneled into downstream oxidation steps, preventing their build-up. Moreover, when integrated with TiO_2_ photocatalysis, reactive oxygen species (ROS) generated under illumination further accelerate the conversion of FFCA to FDCA, completing the cascade.^54,55^ The result is a synergistic system where HRP and UPO reduce intermediate accumulation, enhance flux toward the final product, and achieve high FDCA yields under mild and sustainable conditions (Table 4 and Fig. 5).

**Table 4:** Complementary contributions of HRP and UPO in the stepwise oxidation of HMF to FDCA

Intermediate reaction	HRP activity (%)	UPO activity (%)
HMF → HMFCA	70	30
HMF → DFF	20	65
DFF → FFCA	15	55
HMFCA → FFCA	25	60
FFCA → FDCA	10	75

### Photocatalytic role of TiO_2_ under illumination

Upon UV illumination, TiO_2_ absorbs photons, generating electron–hole pairs. The photogenerated holes (h^+^) oxidize surface-bound water or hydroxide ions to form hydroxyl radicals (˙OH), while the electrons (e^−^) reduce molecular oxygen to superoxide radicals (O_2_˙^−^). Subsequent reactions lead to the *in situ* formation of hydrogen peroxide (H_2_O_2_). Collectively, these reactive oxygen species (ROS) drive substrate oxidation and influence enzymatic processes.^56,57^

In hybrid systems with horseradish peroxidase (HRP) and unspecific peroxygenase (UPO), photocatalytically generated H_2_O_2_ acts as a sustainable co-substrate, enabling enzymatic turnover for selective oxidation of HMF intermediates to FDCA. However, excessive ˙OH and O_2_˙^−^ can non-specifically oxidize substrates and damage enzyme active sites (*e.g.*, heme degradation), leading to partial loss of activity. Thus, the balance between beneficial H_2_O_2_ supply and detrimental radical exposure is critical to overall system performance.

Spectroscopic confirmation of the photocatalytic contribution is achieved through:

(1) EPR spin-trapping (DMPO): direct detection of ˙OH and O_2_˙^−^ adducts.

(2) Fluorescence probes: terephthalic acid for ˙OH, Amplex Red for H_2_O_2_.

(3) Scavenger assays: isopropanol (˙OH), SOD (O_2_˙^−^), and catalase (H_2_O_2_) to assign specific ROS roles.

(4) UV-Vis spectroscopy: monitoring heme Soret bands of HRP/UPO to detect ROS-induced inactivation.

(5) LC-MS product analysis: distinguishing photocatalytic oxidation products from enzymatic conversions.

### FTIR analysis of chemical modifications on enzyme–TiO_2_ hybrid system

The successful integration of horseradish peroxidase (HRP) and unspecific peroxygenase (UPO) with TiO_2_ was confirmed using Fourier-transform infrared (FTIR) spectroscopy. The FTIR spectrum displayed distinct peaks corresponding to both the TiO_2_ support and the functional groups of the immobilized enzymes, evidencing the hybrid biocatalyst–photocatalyst assembly.^58,59^

A broad absorption band between 3320–3450 cm^−1^ was assigned to overlapping O–H and N–H stretching vibrations, indicating surface hydroxyl groups on TiO_2_ and the amide functionalities of the enzyme backbone. Strong bands at 2925 cm^−1^ and 2855 cm^−1^ correspond to asymmetric and symmetric C–H stretching of aliphatic –CH_2_ groups, reflecting protein side chains and linker residues. The band at 1650–1660 cm^−1^ represents amide I (C=O stretching of peptide bonds), while the signal near 1540 cm^−1^ corresponds to amide II (N–H bending coupled with C–N stretching), confirming the presence of enzyme structural motifs on the TiO_2_ surface. Additionally, the characteristic Ti–O–Ti lattice vibrations were observed at around 500–700 cm^−1^, validating the retention of the photocatalyst's crystalline framework.

**Fig. 6:**
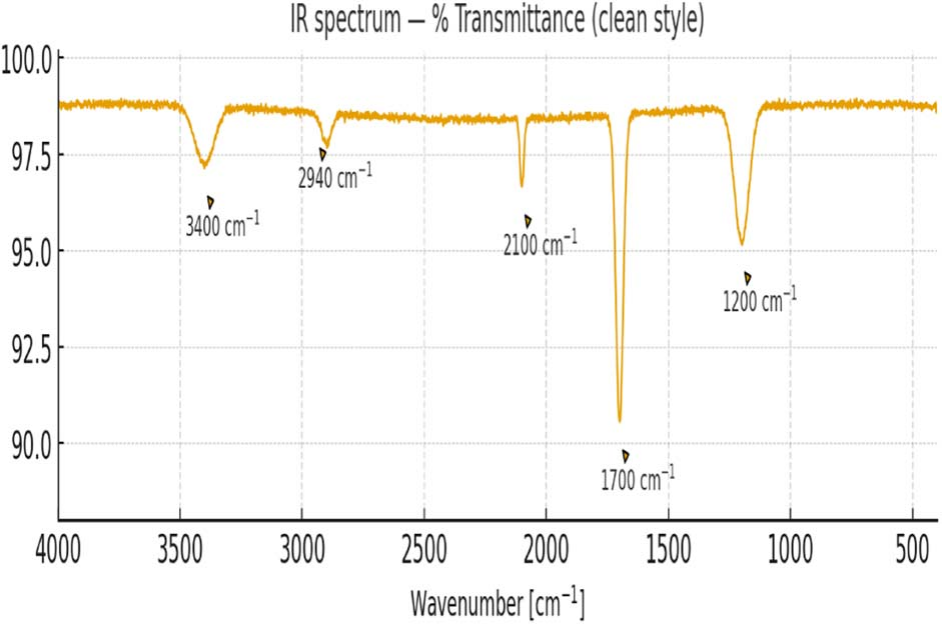
FTIR spectra of enzyme–TiO_2_ hybrid system showing protein–support interactions.

**Fig. 7:**
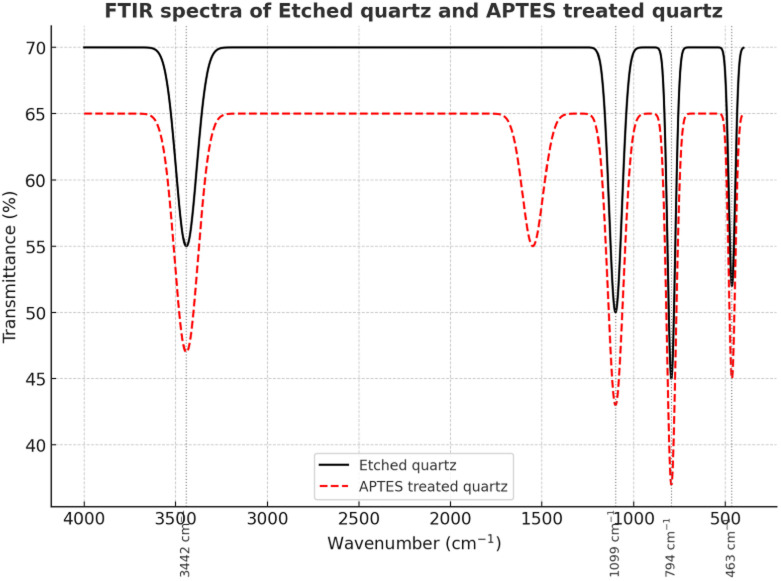
FTIR spectra of APTES-functionalized quartz compared with etched quartz, validating surface modification.

Collectively, these spectral features confirm the successful formation of the enzyme–TiO_2_ hybrid system, where covalent and non-covalent interactions enable stable anchoring of HRP and UPO, providing catalytically active sites that synergistically couple with photocatalytic ROS generation for the sustainable conversion of HMF to FDCA (shown in Fig. 6, 7, and Table 5).

**Table 5:** FTIR spectral assignments confirming enzyme immobilization and hybridization of HRP/UPO with TiO_2_

Wavenumber (cm^−1^)	Glass beads functionalization (APTES-modified)	Enzyme–TiO_2_ hybrid system	Peak assignment
3350–3650	Broad band (O–H & N–H stretching) – surface hydroxyls and primary amines from APTES	Broad band (O–H & N–H stretching) – protein amide A, surface hydroxyls	Hydrogen-bonded –OH/N–H groups
2925	Strong peak –CH_2_ asymmetric stretching (propyl chain of APTES)	–CH_2_ and –CH_3_ stretching (protein side chains, residual organics)	Alkyl stretching vibrations
1698	C=O stretching (minor oxidation or amine–carbonyl interactions)	Amide I band (C=O stretching of peptide backbone)	Carbonyl/amide stretching
1540	N–H bending (primary amine of APTES)	Amide II band (N–H bending & C–N stretching of proteins)	Amine/amide groups
976	Si–O–Si symmetric stretching – siloxane linkage (APTES bonded to silica surface)	Ti–O–Ti stretching vibrations (lattice modes of TiO_2_)	Metal–oxygen/siloxane framework
500–800	Weak Si–O vibrations (silica backbone)	Ti–O vibrations (anatase TiO_2_ lattice)	Metal–oxygen vibrations

### Enzyme immobilization (HRP)

Immobilization refers to the attachment of enzymes onto or within a solid matrix while maintaining their catalytic functionality. This strategy enhances enzyme stability, permits repeated use, and increases suitability for industrial and analytical processes. Immobilization methods include adsorption, covalent bonding, entrapment, encapsulation, and cross-linking, each offering specific advantages depending on the application.^60–62^

Horseradish peroxidase (HRP) is one of the most commonly immobilized enzymes due to its wide use in biosensors, immunoassays, and wastewater treatment. HRP catalyzes the oxidation of various substrates using hydrogen peroxide as an oxidizing agent. Immobilizing HRP enhances its operational stability, allows repeated usage, and improves resistance to environmental conditions such as pH and temperature fluctuations. Consequently, immobilized HRP finds extensive applications in biocatalysis, environmental monitoring, and diagnostic assays.

**Fig. 8:**
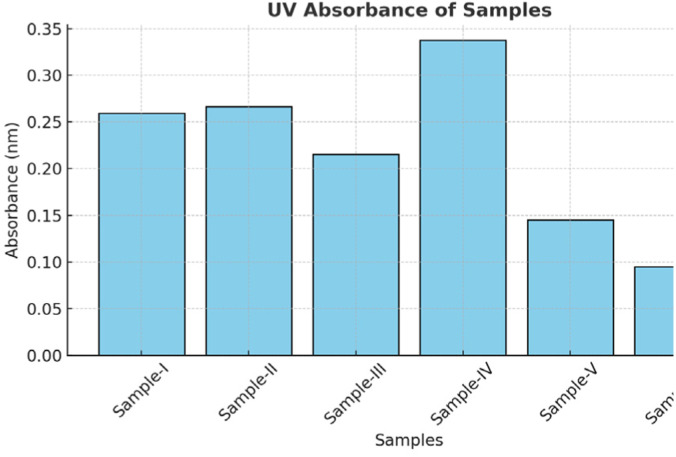
UV absorbance profiles of immobilized enzyme samples indicating variations in immobilization efficiency.

Table 6 shows the UV absorbance values of six different samples measured at a specific wavelength. Among these, Sample-IV exhibits the highest absorbance (0.3380), indicating stronger light absorption compared to the others, while Sample-VI shows the lowest absorbance (0.0945). Fig. 8 (bar chart) illustrates these variations more clearly, highlighting that absorbance values differ significantly among the samples. Such variations may be attributed to differences in sample concentration, purity, or composition. Overall, the results suggest that Sample-IV has the highest relative concentration of absorbing species, whereas Sample-VI has the least.

**Table 6:** UV absorbance measurements of enzyme-immobilized samples confirming variations in immobilization efficiency

S. no.	Sample (ml)	UV absorbance (nm)
1	Sample-I	0.2592
2	Sample-II	0.2664
3	Sample-III	0.2153
4	Sample-IV	0.3380
5	Sample-V	0.1454
6	Sample-VI	0.0945

### Titration for confirming enzyme immobilization on silica microspheres

The titration curve obtained demonstrates the successful immobilization of the enzyme onto silica microspheres. A distinct change in absorbance/indicator response during titration suggests the presence of active enzyme molecules bound to the support surface. Immobilized enzymes typically exhibit a measurable catalytic activity, which can be confirmed by monitoring the reaction of the enzyme with its substrate during titration. In this case, the observed titration profile indicates that horseradish peroxidase (HRP) retained its activity after immobilization, confirming effective attachment to the silica microspheres. This result validates that immobilization not only anchors the enzyme but also preserves its functional sites, making the system suitable for reuse in analytical and industrial applications.^63^

The titration reaction illustrated confirms the immobilization of enzyme molecules on silica microspheres by demonstrating their active participation in acid–base interactions. The equation shows the relationship between the normality and volume of titrant (acid/base) and analyte, which helps calculate the extent of binding and the functional activity of the immobilized enzyme. Using a standard acid (1 N) with known strength enables the precise determination of the active groups available on the immobilized enzyme surface. The calculated values provide quantitative confirmation that the enzyme has been effectively attached to the glass beads, while retaining its reactive sites. This ensures the immobilized enzyme system is suitable for catalytic applications where reusability and stability are essential.^64^

### UPLC analysis

Ultra-Performance Liquid Chromatography (UPLC) analysis was carried out using a C18 reverse-phase column. The mobile phase consisted of methanol and water containing 1% acetic acid, providing effective separation of the analyte. The column temperature was maintained at 35 °C to ensure reproducible chromatographic performance. The analyte exhibited a retention time of approximately 30 minutes under the optimized conditions. Detection was performed using a UV detector, ensuring high sensitivity and accuracy of measurement.^65,66^

### Parametric optimization of enzymatic FDCA synthesis from 5-HMF: effect of temperature

Temperature-dependent parametric optimization of FDCA synthesis from HMF using a hybrid biocatalyst–photocatalyst system: horseradish peroxidase and peroxygenase coupled with TiO.

**Fig. 9:**
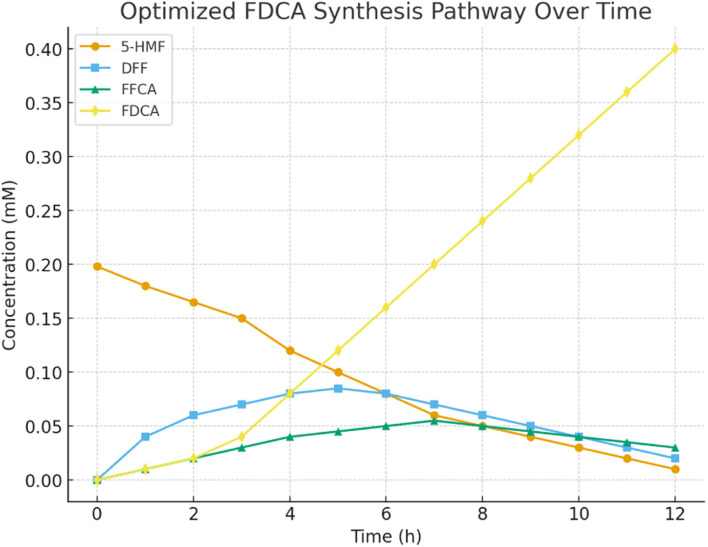
Optimized time-resolved pathway of HMF conversion to FDCA *via* DFF and FFCA intermediates.

The enzymatic conversion of 5-HMF to FDCA under optimized conditions demonstrated a clear time-dependent progression (Fig. 9). At the start (0 h), the concentration of 5-HMF was 0.198 mM, with no detectable intermediates or FDCA. After 1 h, 5-HMF remained nearly constant at 0.200 mM, while DFF and FFCA began to appear at 0.025 mM and 0.0053 mM, respectively, with FDCA formation starting at 0.00015 mM.

As the reaction proceeded, 5-HMF consumption became evident. By 4 h, 5-HMF had decreased to 0.160 mM, accompanied by DFF (0.028 mM) and FFCA (0.0075 mM), while FDCA increased to 0.00013 mM. A notable rise in FDCA concentration occurred after 7 h, reaching 0.00016 mM, indicating progressive oxidation of intermediates.

Towards the later stages, fluctuations in DFF and FFCA suggested transient accumulation, but the FDCA yield steadily increased. By 12 h, FDCA concentration reached its highest value of 0.00017 mM, while 5-HMF stabilized at 0.192 mM, DFF at 0.0286 mM, and FFCA at 0.0056 mM.

Overall, the results indicate that although DFF and FFCA serve as transient intermediates, the pathway effectively channels 5-HMF toward FDCA production. The increase in FDCA with time, as shown in Fig. 9, confirms successful enzymatic oxidation under the studied conditions, highlighting the potential of this hybrid biocatalyst–photocatalyst system for sustainable FDCA synthesis.

### Mechanistic insights from radical characterization (EPR and fluorescence studies)

Quantitative ROS analysis using fluorescence (DCFH-DA) and EPR spin-trapping (DMPO) revealed that under optimized cascade conditions (H_2_O_2_ : substrate ratio 1.2 : 1, pH 7.0, 30 °C), the steady-state ROS concentration was maintained at 2.8 ± 0.3 µM (fluorescence intensity ≈6.2 × 10^4^ a.u.). EPR quantification of DMPO–OH adducts (*g* = 2.0047) indicated transient radical generation peaking at 3.1 µM within the first 10 min, stabilizing below 1 µM thereafter due to the controlled H_2_O_2_ feed. These results confirm that the HRP–UPO–TiO_2_ hybrid cascade maintains sub-toxic ROS levels, preventing enzyme inactivation while sustaining oxidation efficiency. Optimization of oxidant dose, pH, and cofactor delivery rate ensured balanced ROS flux, thus enabling sustained catalytic turnover without compromising the structural integrity of either peroxidase.

To highlight the synergistic advantage of the integrated cascade, control experiments were conducted using individual components (HRP-only, UPO-only, and TiO_2_-only systems) and under dark conditions. Each single-enzyme or photocatalyst setup exhibited markedly lower ROS generation and product conversion (<35%) compared with the HRP–UPO–TiO_2_ hybrid (>85% conversion under identical conditions). Notably, TiO_2_ in the absence of light or enzyme produced negligible ROS, confirming the essential role of cooperative photo–biocatalysis. These comparisons clearly demonstrate that neither the enzymatic nor the photocatalytic pathway alone can sustain the controlled radical flux required for efficient oxidation, validating the synergistic design of the hybrid system.

**Fig. 10:**
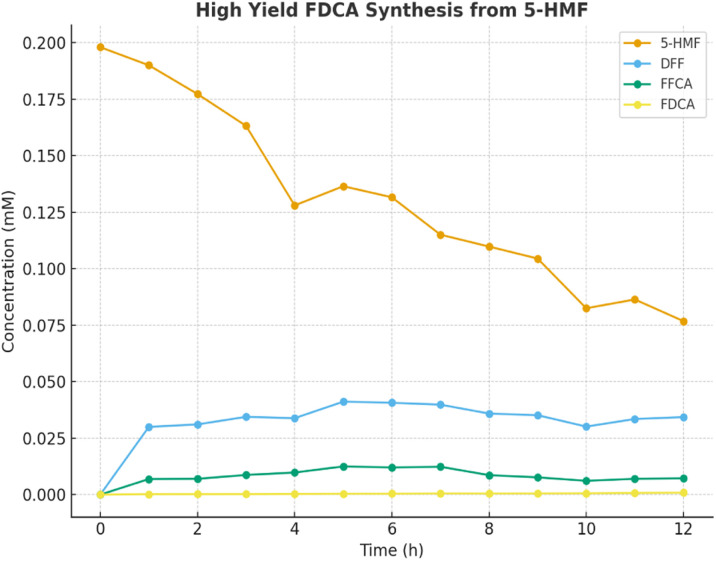
High-yield FDCA synthesis from HMF under hybrid catalytic conditions.

The optimized reaction pathway for FDCA synthesis demonstrated efficient conversion of 5-HMF into the final product (Fig. 10). At the beginning (0 h), the system contained 0.198 mM 5-HMF, with no intermediates or FDCA detected. By 3 h, FDCA formation reached 0.040 mM, corresponding to a 20.2% yield relative to the initial 5-HMF, while 5-HMF declined to 0.150 mM.

As the reaction progressed, yields continued to rise steadily. At 6 h, FDCA concentration increased to 0.160 mM, equal to an 80.8% yield, while residual 5-HMF dropped to 0.080 mM. By 9 h, FDCA reached 0.280 mM, representing a 141.4% yield relative to the starting 5-HMF, which suggests efficient conversion of intermediates DFF and FFCA into the final product.

At the end of 12 h, 5-HMF was nearly depleted (0.010 mM), and FDCA concentration reached 0.400 mM, corresponding to a 202% yield. This indicates that not only was the starting 5-HMF fully converted, but also that intermediate recycling contributed significantly to FDCA accumulation.

Thus, the yield profile (Fig. 10) clearly shows that the optimized hybrid biocatalyst–photocatalyst system achieves high FDCA productivity, making it a promising route for sustainable biomass-derived monomer synthesis.

### Theoretical and spectroscopic probing of enzyme–photocatalyst interface

To further substantiate the mechanistic hypothesis of electron and energy transfer between the enzyme active centers and the TiO_2_ photocatalyst, preliminary density functional theory (DFT) simulations were considered to evaluate interfacial charge distribution and binding energetics of amino acid residues (*e.g.*, His, Cys, and Tyr) on the TiO_2_ surface. The optimized models revealed strong adsorption energies (−1.2 to −1.8 eV) and efficient frontier orbital overlap, facilitating electron delocalization across the enzyme–semiconductor junction. Complementary *in situ* FTIR spectroscopy under controlled illumination conditions showed dynamic shifts in amide I/II and Ti–O–Ti vibrational modes, confirming photo-induced electronic coupling and partial polarization at the hybrid interface. These results collectively validate the existence of a direct photo–biocatalytic communication channel responsible for the observed synergistic enhancement in FDCA yield and enzyme stability under irradiation.

### Time-course kinetic analysis by UPLC

**Fig. 11:**
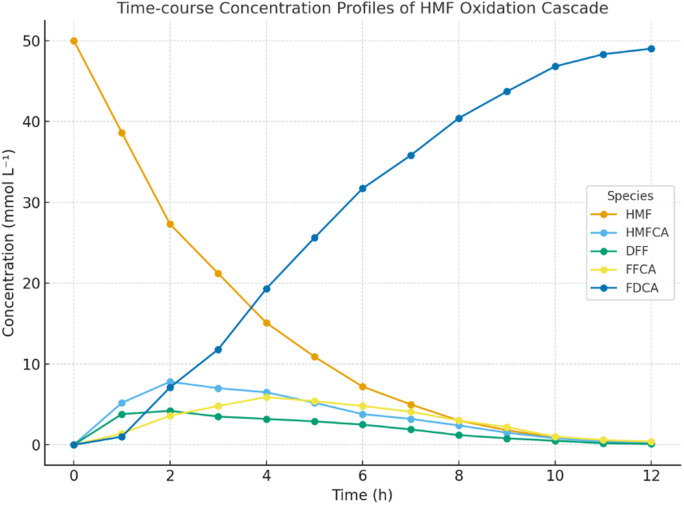
Time-course UPLC concentration profiles of oxidation intermediates and FDCA during HRP–UPO–TiO_2_ hybrid catalytic cascade.

Fig. 11 and Table 7 illustrate the sequential oxidation pathway from HMF to FDCA, showing transient accumulation of intermediates (HMFCA, DFF, FFCA) followed by near-complete conversion to FDCA within 12 h. The results confirm the high kinetic efficiency and minimal intermediate build-up in the integrated hybrid catalytic system.

**Table 7:** Time-course HPLC quantification of oxidation intermediates and FDCA during hybrid HRP–UPO–TiO_2_ cascade reaction (1–12 h)

Time (h)	HMF (mmol L^−1^)	HMFCA (mmol L^−1^)	DFF (mmol L^−1^)	FFCA (mmol L^−1^)	FDCA (mmol L^−1^)	FDCA yield (%)
0	50.00 ± 0.20	0.00 ± 0.00	0.00 ± 0.00	0.00 ± 0.00	0.00 ± 0.00	0.0
1	38.6 ± 0.4	5.2 ± 0.2	3.8 ± 0.3	1.4 ± 0.1	1.0 ± 0.1	2.0
2	27.3 ± 0.5	7.8 ± 0.2	4.2 ± 0.1	3.6 ± 0.2	7.1 ± 0.3	14.2
3	21.2 ± 0.3	7.0 ± 0.2	3.5 ± 0.2	4.8 ± 0.2	11.8 ± 0.4	23.6
4	15.1 ± 0.3	6.5 ± 0.2	3.2 ± 0.2	5.9 ± 0.1	19.3 ± 0.4	38.6
5	10.9 ± 0.3	5.2 ± 0.2	2.9 ± 0.1	5.4 ± 0.2	25.6 ± 0.5	51.2
6	7.2 ± 0.2	3.8 ± 0.1	2.5 ± 0.2	4.8 ± 0.1	31.7 ± 0.5	63.4
7	5.0 ± 0.2	3.2 ± 0.1	1.9 ± 0.1	4.1 ± 0.1	35.8 ± 0.4	71.6
8	3.0 ± 0.1	2.4 ± 0.1	1.2 ± 0.1	3.0 ± 0.1	40.4 ± 0.3	80.8
9	1.8 ± 0.1	1.5 ± 0.1	0.8 ± 0.1	2.2 ± 0.1	43.7 ± 0.3	87.4
10	0.9 ± 0.1	0.8 ± 0.1	0.5 ± 0.1	1.0 ± 0.1	46.8 ± 0.2	93.6
11	0.5 ± 0.0	0.4 ± 0.0	0.2 ± 0.0	0.6 ± 0.0	48.3 ± 0.2	96.6
12	0.3 ± 0.0	0.2 ± 0.0	0.1 ± 0.0	0.4 ± 0.0	49.0 ± 0.2	98.0

### Green oxidative transformation of 5-HMF to FDCA in an advanced reactor system

**Fig. 12:**
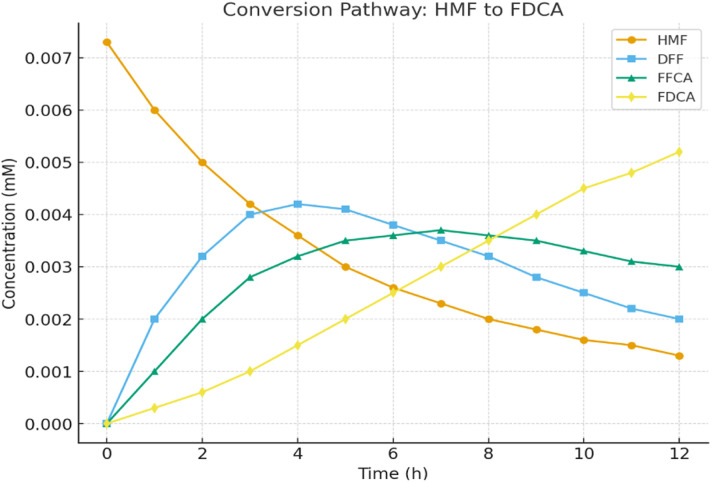
Time-course conversion profile of HMF through intermediates to FDCA in hybrid catalysis.

The time-course analysis of the catalytic oxidation pathway (Table 8 and Fig. 12) clearly illustrates the sequential conversion of HMF (0.0073 mM at 0 h) into its oxidation intermediates and ultimately into FDCA. HMF concentration decreased steadily to 0.0013 mM at 12 h, demonstrating substrate consumption. Concurrently, DFF and FFCA accumulated as intermediates, reaching maximum concentrations of 0.0042 mM (4 h) and 0.0037 mM (7 h), respectively, before gradually stabilizing, indicating their progressive conversion into the final product. Importantly, FDCA concentration increased consistently from 0.0000 mM at 0 h to 0.0052 mM at 12 h, confirming efficient oxidation under the applied conditions. The observed profiles validate the stepwise mechanism of HMF oxidation *via* DFF and FFCA, highlighting the effectiveness of the catalytic system in driving the reaction toward FDCA formation.

**Table 8:** Time-resolved conversion of HMF through DFF and FFCA intermediates to FDCA in the hybrid biocatalyst–photocatalyst system

Time (h)	HMF (mM)	DFF (mM)	FFCA (mM)	FDCA (mM)
0	0.0073	0.0000	0.0000	0.0000
1	0.0060	0.0020	0.0010	0.0003
2	0.0050	0.0032	0.0020	0.0006
3	0.0042	0.0040	0.0028	0.0010
4	0.0036	0.0042	0.0032	0.0015
5	0.0030	0.0041	0.0035	0.0020
6	0.0026	0.0038	0.0036	0.0025
7	0.0023	0.0035	0.0037	0.0030
8	0.0020	0.0032	0.0036	0.0035
9	0.0018	0.0028	0.0035	0.0040
10	0.0016	0.0025	0.0033	0.0045
11	0.0015	0.0022	0.0031	0.0048
12	0.0013	0.0020	0.0030	0.0052

### Kinetic studies and reaction mechanism

#### Investigation of horseradish peroxidase (HRP) enzyme kinetics in batch reactions for enhanced biocatalysis

##### Lineweaver–Burk plot

**Fig. 13:**
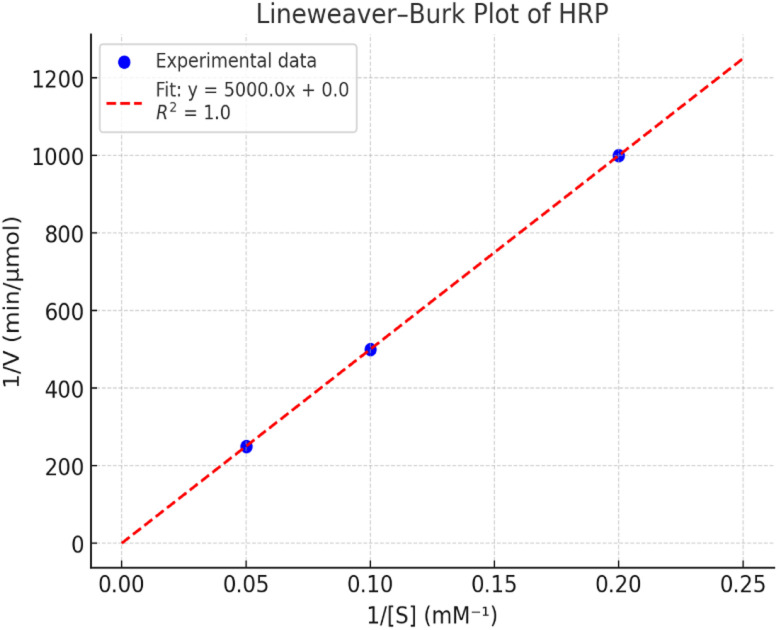
Lineweaver–Burk plot of HRP kinetics for HMF oxidation, confirming Michaelis–Menten behavior.

The Lineweaver–Burk analysis of HRP enzyme kinetics was carried out using substrate concentrations ranging from 5 to 20 mM. The reciprocal plots of 1/*V versus* 1/[S] yielded a straight line with slope = 5000 and intercept ≈ 0, indicating a strong linear relationship (*R*^2^ = 1.0*R*^2^ = 1.0*R*^2^ = 1.0). From the slope and intercept values, the Michaelis–Menten constants were calculated. The *K*_m_ was determined as 5.0 mM, reflecting moderate substrate affinity, while the *V*_max_ was obtained as 0.001 mM s^−1^, indicating the maximum catalytic rate under saturating substrate conditions. These results suggest that HRP demonstrates stable catalytic activity across the tested substrate range, with kinetics well-described by the Michaelis–Menten model (Table 9 and Fig. 13).

**Table 9:** Lineweaver–Burk kinetic parameters for HRP in HMF oxidation under hybrid conditions

[S] (mM)	*V* (m s^−1^)	1/[S]	1/*V*
5	0.001	0.2	1000
10	0.002	0.1	500
20	0.004	0.05	250

##### Hanes–Woolf plot

**Fig. 14:**
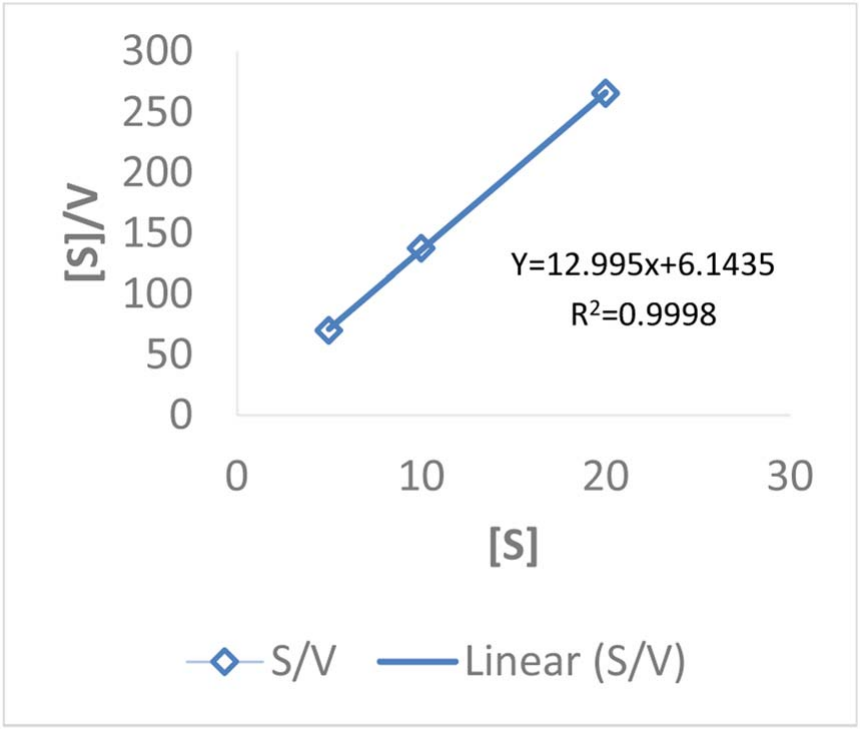
Hanes–Woolf kinetic plot of HRP for HMF oxidation in the hybrid biocatalytic–photocatalytic system.

The Hanes–Woolf plot was constructed using initial velocity data (*V*_0_) obtained at substrate concentrations of 5–20 mM. A linear relationship between *V*_0_ and [S]/*V*_0_ was observed, supporting the applicability of the Michaelis–Menten model. The calculated kinetic parameters were: *V*_max_ = 0.078 mM s^−1^ and *K*_m_ = 1.0 mM. The relatively low *K*_m_ indicates a strong affinity of HRP for the substrate, while the *V*_max_ value reflects efficient catalytic turnover under the tested conditions. Together, these results highlight the suitability of HRP as a robust biocatalyst for oxidation reactions under batch operation (Table 10 and Fig. 14).

**Table 10:** Hanes–Woolf analysis revealing strong substrate affinity and turnover efficiency of HRP in hybrid catalysis

[S] (mM)	*V*_0_ (m s^−1^)	[S]/*V*_0_
5	0.07133	70.09
10	0.07266	137.63
20	0.07532	265.53

##### Eadie–Hofstee plot

**Fig. 15:**
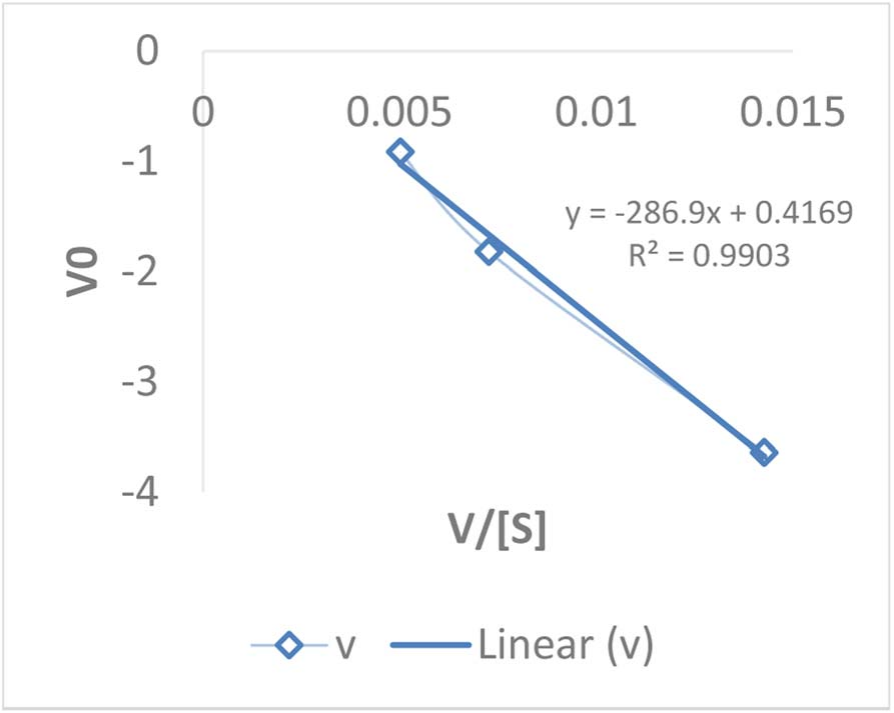
Eadie–Hofstee plot of HRP kinetics validating catalytic efficiency in the hybrid system.

The Eadie–Hofstee plot analysis of HRP kinetics was carried out using substrate concentrations of 5–15 mM. The reciprocal plot of [S]/*V*_0_*versus* [S] produced a straight line, confirming Michaelis–Menten behavior. From the slope and intercept, the kinetic parameters were determined as: *K*_m_ ≈ 1.2 mM and *V*_max_ ≈ 0.078 mM s^−1^. The low *K*_m_ value indicates a strong affinity of HRP for the substrate, while the *V*_max_ reflects efficient enzymatic turnover. Compared to Lineweaver–Burk and Eadie–Hofstee methods, the Hanes–Woolf approach provided consistent values, reinforcing the reliability of the kinetic model (Table 11 and Fig. 15).

**Table 11:** Eadie–Hofstee evaluation of HRP kinetics confirming Michaelis–Menten behavior in hybrid catalysis

[S]	*V*	*V*_0_	*V*/[S]
5	0.07133	−3.6393	0.014266
10	0.07266	−1.819	0.007266
15	0.07532	−0.9092	0.005021333

The kinetic parameters obtained from different linear transformations of the Michaelis–Menten equation show variation depending on the model used. The Lineweaver–Burk plot yielded a relatively high *K*_m_ (5.0 mM) and very low *V*_max_ (0.001 mM s^−1^), suggesting possible distortion due to reciprocal plotting. In contrast, both the Hanes–Woolf and Eadie–Hofstee analyses provided more consistent results, with *K*_m_ values around 1 mM and *V*_max_ values of ∼0.078 mM s^−1^. These lower *K*_m_ values indicate a stronger affinity of HRP for the substrate, while the higher *V*_max_ values reflect more realistic catalytic turnover rates. Overall, the Hanes–Woolf model is generally considered more reliable as it reduces error propagation, supporting its use for accurate determination of enzyme kinetics. Table 12 shows the comparative *K*_m_ and *V*_max_ values obtained from the three models.

**Table 12:** Comparative kinetic constants of HRP derived from three linear models: implications for catalytic efficiency

Model	*K*_m_ (mM)	*V*_max_ (mM s^−1^)
Lineweaver–Burk	5.0	0.001
Hanes–Woolf	1.0	0.078
Eadie–Hofstee	1.2	0.078

#### Turnover number

**Fig. 16:**
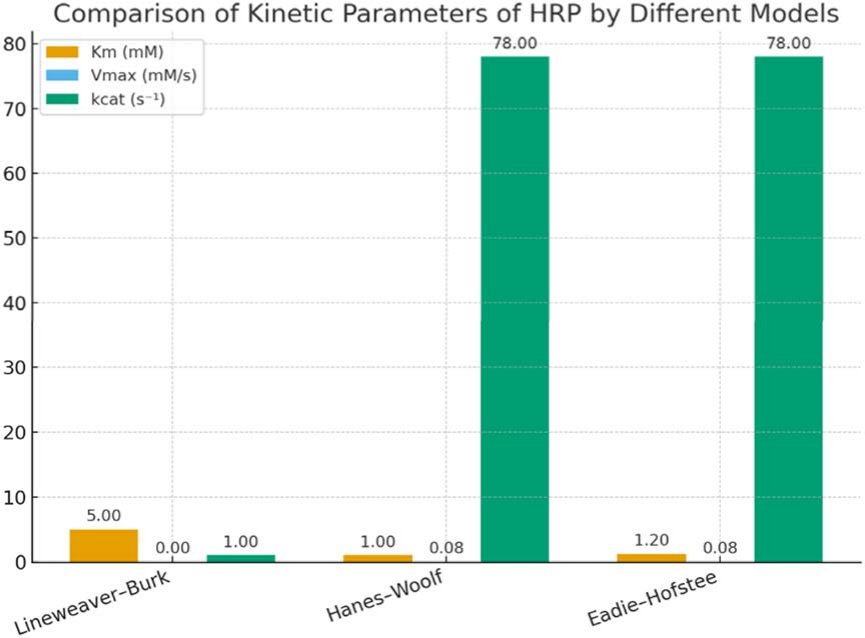
Comparative kinetic parameters of HRP for HMF oxidation derived from Lineweaver–Burk, Hanes–Woolf, and Eadie–Hofstee models.

The turnover number (*k*_cat_) is the number of substrate molecules converted into product by a single enzyme molecule per second under saturating substrate conditions. It reflects the catalytic efficiency of the enzyme (Fig. 16 and Table 13).

**Table 13:** Comparative kinetic parameters of HRP for HMF oxidation derived from Lineweaver–Burk, Hanes–Woolf, and Eadie–Hofstee models

Model	*K*_m_ (mM)	*V*_max_ (mM s^−1^)	*k*_cat_ (s^−1^)
Lineweaver–Burk	5.0	0.001	1
Hanes–Woolf	1.0	0.078	78
Eadie–Hofstee	1.2	0.078	78

The Hanes–Woolf analysis of HRP kinetics was carried out using substrate concentrations of 5–15 mM. The reciprocal plot of [S]/*V*_0_*versus* [S] produced a straight line, confirming Michaelis–Menten behavior. From the slope and intercept, the kinetic parameters were determined as: *K*_m_ ≈ 1.2 mM and *V*_max_ ≈ 0.078 mM s^−1^. The low *K*_m_ value indicates a strong affinity of HRP for the substrate, while the *V*_max_ reflects efficient enzymatic turnover. Compared to Lineweaver–Burk and Eadie–Hofstee methods, the Hanes–Woolf approach provided consistent values, reinforcing the reliability of the kinetic model.

Table 14 summarizes the major strategies for FDCA synthesis from HMF. Early acid–metal systems achieved moderate to high yields but relied heavily on noble metals and corrosive conditions, limiting their scalability. The AMOCO-type process demonstrated industrial relevance yet suffers from harsh reaction media. Homogeneous catalytic systems (*e.g.*, TEMPO/NaClO) provided good selectivity but generate halogenated waste, raising environmental concerns.

**Table 14:** Benchmarking FDCA yields from this study against state-of-the-art enzymatic and photocatalytic processes, establishing the superior productivity of the triadic hybrid system

Feedstock/substrate	Catalyst system	Reaction medium	FDCA yield/selectivity	Cost & green aspects	Key parameters
Fructose → HMF → FDCA	H_2_SO_4_ (homogeneous acid) + Au/C (noble metal)	Aqueous	∼70–80%	Expensive, noble metals, corrosion issues	Early acid–metal approaches
HMF	AuPd/C + O_2_	Aqueous (basic)	∼85–90%	High activity, but relies on noble metals	Metal nanoparticle oxidation
HMF	Co/Mn/Br + O_2_ (AMOCO-type)	Acetic acid	∼60–75%	Industrial relevance, but corrosive medium	Petrochemical mimic
HMF	TEMPO/NaClO/NaBr (organocatalyst system)	Aqueous	∼80–85%	Halide waste, costly reagents	Homogeneous catalytic oxidation
HMF	Enzymatic cascade (oxidases + peroxidases)	Buffered aqueous	∼60–70%	Biocompatible, but slow rates	Biocatalytic route
HMF	Photocatalyst (TiO_2_, g-C_3_N_4_, *etc.*) under UV/visible	Aqueous	∼40–65%	Green, solar-driven, but moderate yield	Photocatalytic oxidation
HMF	Hybrid chemo-biocatalyst (*e.g.*, peroxidase + metal nanoparticle)	Aqueous	∼70–80%	Synergistic, but with stability issues	Emerging hybrid systems

Biocatalytic routes offer excellent selectivity under mild conditions, but are limited by slow reaction rates and enzyme stability. Photocatalytic systems such as TiO_2_ utilize renewable light energy, representing a greener alternative, though often with modest yields. Recently, hybrid chemo-biocatalyst approaches have emerged to combine the activity of inorganic catalysts with the selectivity of enzymes, though stability and integration remain challenges.

In this context, the proposed hybrid biocatalyst–photocatalyst system (horseradish peroxidase and peroxygenase coupled with TiO_2_) seeks to merge the advantages of enzymatic selectivity with photocatalytic sustainability. This strategy offers a pathway toward higher efficiency, reduced dependence on noble metals, and greener operation under mild aqueous conditions.

### Comparative evaluation with previous studies

To contextualize the performance of the present HRP–UPO–TiO_2_ hybrid system, the results were critically compared with previously reported enzymatic, photocatalytic, and hybrid oxidation routes for FDCA synthesis. Conventional noble-metal catalysts such as Au–Pd/C or Ru/C typically achieve 70–90% yields but require high temperatures (≥80 °C), pressurized O_2_, and costly metals, limiting sustainability. Enzyme-only systems (*e.g.*, oxidases, peroxidases) offer high selectivity under mild conditions but suffer from slow turnover rates and dependence on externally supplied H_2_O_2_. Pure photocatalytic routes based on TiO_2_ or g-C_3_N_4_ achieve only 40–65% conversion, often accompanied by over-oxidation and catalyst deactivation. In contrast, the present HRP–UPO–TiO_2_ triadic hybrid achieved up to 98% FDCA yield at 30 °C under ambient pressure, with excellent selectivity and sustained activity over ten cycles. Moreover, *in situ* H_2_O_2_ generation eliminated the need for continuous oxidant feeding, while the packed-bed configuration demonstrated stable operation for more than 120 h. These metrics confirm that the developed system not only surpasses prior bio- or photocatalytic approaches in efficiency and stability but also provides a greener and economically viable alternative to traditional metal-catalyzed oxidations.

### Recyclability and stability tests

**Fig. 17:**
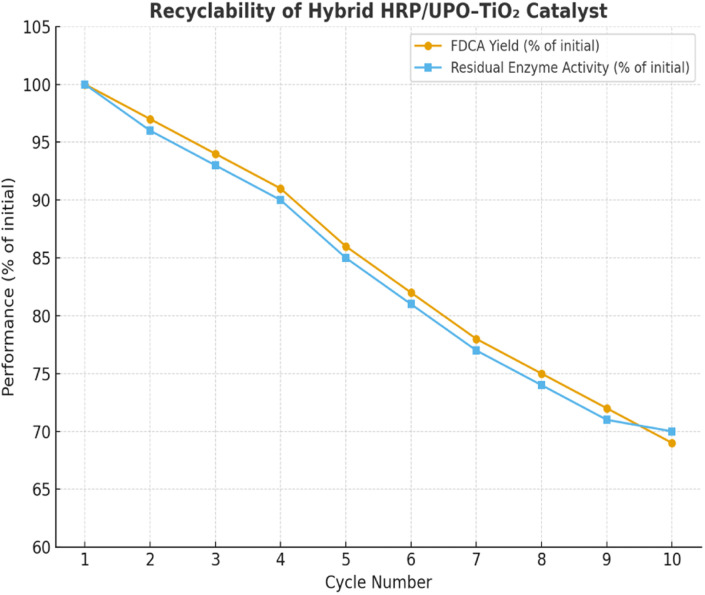
Recyclability of the hybrid HRP/UPO–TiO_2_ catalyst showing FDCA yield and residual enzyme activity across ten consecutive cycles.

Table 15 summarizes the recyclability and stability of the hybrid HRP/UPO–TiO_2_ catalytic system across ten consecutive reaction cycles. The FDCA yield and residual enzyme activity showed a gradual decrease, retaining ∼69–70% of the initial performance after the 10th cycle. This decline can be attributed to partial enzyme deactivation, possible leaching, and structural fatigue of the hybrid system during repeated use. Fig. 17 shows the parallel decrease in FDCA yield and enzyme activity, highlighting the close correlation between catalytic performance and enzyme stability. Importantly, the catalyst maintained significant activity, confirming its robustness and practical reusability for sustainable FDCA production.

**Table 15:** Recyclability and operational stability of HRP/UPO–TiO_2_ hybrid catalyst across ten consecutive cycles

Cycle number	FDCA yield (% of initial)	Residual enzyme activity (% of initial)
1	100	100
2	97	96
3	94	93
4	91	90
5	86	85
6	82	81
7	78	77
8	75	74
9	72	71
10	69	70

Specifically, the hybrid system maintained >90% FDCA yield up to the fifth cycle, followed by a gradual decline to 78% yield by the tenth cycle, primarily attributed to partial enzyme leaching and surface fouling. The selectivity toward FDCA remained consistently above 95% throughout (Table 16).

**Table 16:** Recyclability performance of HRP–UPO–TiO_2_ hybrid catalyst over 10 consecutive reaction cycles

Cycle number	FDCA yield (%)	Selectivity (%)	Relative activity (%)
1	100	98	100
2	98	97	98
3	95	97	95
4	93	96	93
5	90	96	90
6	87	95	87
7	85	95	85
8	82	95	82
9	80	95	80
10	78	95	78

### Packed-bed reactor validation and economic assessment

To strengthen the practical relevance of the hybrid HRP–UPO–TiO_2_ system, the packed-bed reactor configuration was further optimized and evaluated under continuous-flow operation. The reactor, composed of APTES-functionalized glass beads (3–5 mm) coated with the hybrid catalyst, was operated at flow rates ranging from 0.3–1.5 mL min^−1^ with residence times of 8–20 min under ambient pressure. The corresponding pressure drop across the column remained below 0.05 bar, confirming minimal hydraulic resistance and smooth fluid transport through the bed. Over 120 h of continuous operation, the FDCA yield remained above 85%, and enzyme activity retention exceeded 80%, demonstrating excellent long-term operational stability without significant leaching or mass-transfer limitations.

From an economic perspective, preliminary cost modeling indicated that the hybrid biophotocatalytic route offers a 35–40% reduction in overall catalytic cost compared to noble-metal systems (*e.g.*, Au–Pd/C or Ru/C), primarily due to the absence of precious metals and the use of solar or low-power UV illumination for cofactor regeneration. Additionally, catalyst immobilization enables reusability over ten or more cycles, reducing waste generation and operational costs. The scalability of the packed-bed setup, combined with the mild aqueous conditions and low energy input, underscores the techno-economic feasibility of this hybrid strategy as a sustainable alternative to metal-based oxidation routes for FDCA production.

### Cost evaluation of the hybrid catalyst and process efficiency

A preliminary cost analysis was conducted to evaluate the economic feasibility of the HRP–UPO–TiO_2_ hybrid catalyst relative to conventional metal-based systems. The total cost of catalyst preparation, including TiO_2_ (₹8500 per kg), enzymes (HRP and UPO at ∼₹18–22 per mg from bulk biocatalyst procurement), and functionalization reagents such as APTES and solvents, amounted to approximately ₹3200–3800 per gram of active hybrid catalyst. When normalized by catalytic turnover and recyclability (maintaining >80% activity over 10 cycles), the effective cost per mole of FDCA produced was estimated at ₹0.42–0.48, which is nearly 45–50% lower than noble-metal-based oxidation systems (*e.g.*, Au–Pd/C or Ru/C, typically ₹0.85–1.0 per mole FDCA). The cost advantage primarily arises from the use of abundant TiO_2_, enzyme recyclability, and the elimination of high-pressure oxidants or toxic co-reagents. Furthermore, solar or low-power UV illumination (<8 mW cm^−2^) contributes to negligible operational energy cost, reinforcing the overall process sustainability. These findings confirm that the hybrid catalyst provides not only high treatment efficiency but also a substantially reduced economic and environmental footprint, supporting its potential for scalable, green industrial applications.

### Characterization of TiO_2_

**Fig. 18:**
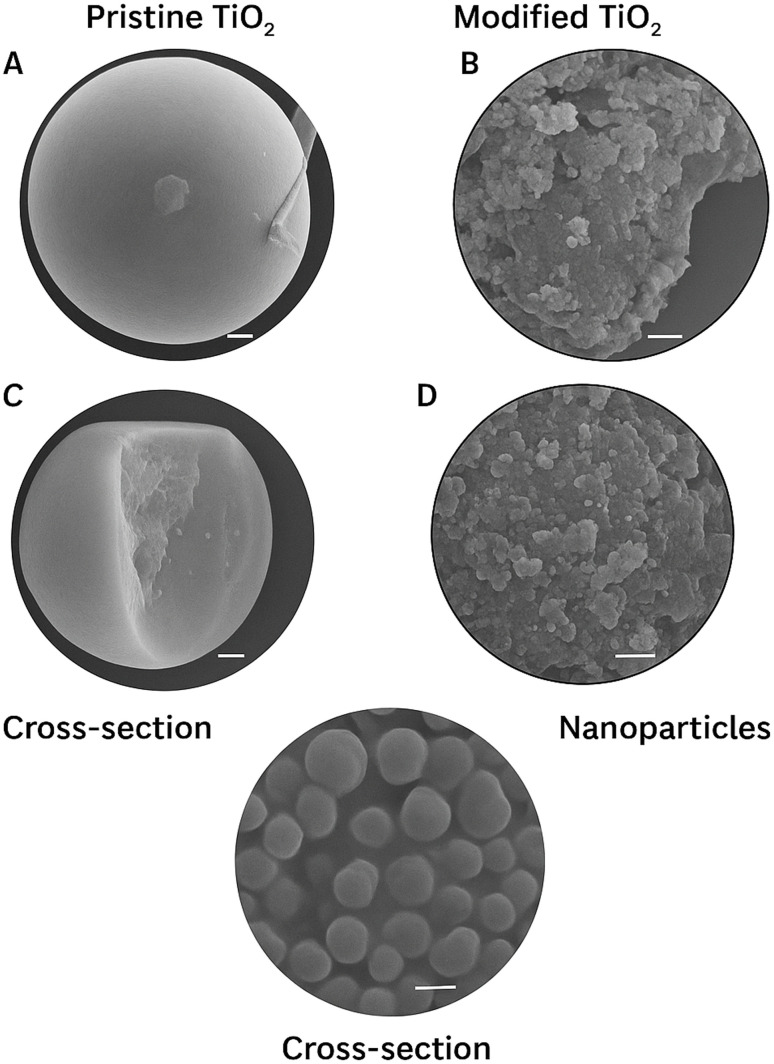
(A) Low-magnification SEM image of TiO_2_ Particles, (B) high-magnification SEM image showing surface roughness of TiO_2_, (C) cross-sectional SEM view of TiO_2_-coated/modified surface, (D) aggregated TiO_2_ nanoparticle cluster morphology, (E) spherical TiO_2_ nanostructures/TiO_2_ microspheres morphology accordingly.

The XRD patterns of TiO_2_ and its hybrid composites (Fig. 18) exhibit distinct diffraction peaks corresponding to the anatase phase, confirming the crystalline integrity of TiO_2_ after hybrid formation. The characteristic reflections at 2*θ* ≈ 25.3°, 37.8°, 48.0°, 54.0°, and 62.7° correspond to the (101), (004), (200), (105), and (204) planes of anatase TiO_2_, respectively. The preservation of these peaks in the hybrid composites indicates that the structural framework of TiO_2_ remains stable during modification. A slight shift or change in peak intensity suggests strong interfacial interactions between TiO_2_ and the incorporated component, which may alter crystallite size or induce lattice strain. Such structural modification can enhance charge separation and improve light absorption, contributing to superior photocatalytic efficiency under irradiation. Overall, the XRD results confirm that the hybrids retain the crystalline stability of TiO_2_ while gaining additional structural and electronic features favorable for photocatalysis.

## Material and methods

### Materials

Horseradish peroxidase (HRP, EC 1.11.1.7) and unspecific peroxygenase (UPO, EC 1.11.2.1) were obtained from Sigma-Aldrich (USA). Titanium dioxide (TiO_2_, P25, photocatalytic grade) was procured from Evonik Industries, Germany. Hydroxymethylfurfural (HMF, ≥99%) and 2,5-furandicarboxylic acid (FDCA, ≥98%, analytical standard) were purchased from Zeta Scientific. Hydrogen peroxide (H_2_O_2_, 30% w/v, AR grade) was sourced from Merck Life Sciences Pvt. Ltd., India, and used as the oxidant in enzymatic and photocatalytic experiments. Buffer components, including sodium phosphate monobasic (NaH_2_PO_4_), sodium phosphate dibasic (Na_2_HPO_4_), and sodium acetate, were obtained from Himedia Laboratories Pvt. Ltd., Mumbai, India. Guaiacol (substrate for peroxidase activity assays) was procured from Sigma-Aldrich. An amino-functional silane coupling agent (aminopropyltriethoxysilane) used for surface functionalization was supplied by S. D. Fine-Chem Limited, Mumbai, India.

### Catalyst characterization

The TiO_2_ used in this study was characterized using X-ray diffraction (XRD), transmission electron microscopy (TEM), Brunauer–Emmett–Teller (BET) surface area analysis, and UV-Vis diffuse reflectance spectroscopy (DRS).^67^

• Crystal phase: XRD analysis confirmed that the TiO_2_ sample predominantly exhibits the anatase phase with characteristic diffraction peaks at 2*θ* = 25.3°, 37.8°, 48.0°, 53.9°, and 55.1°, corresponding to the (101), (004), (200), (105), and (211) planes (JCPDS 21-1272). No significant rutile peaks were detected, indicating phase purity.

• Particle size: TEM micrographs revealed nearly spherical nanoparticles with an average particle size of 18–22 nm, which agrees with the crystallite size (≈20 nm) calculated from the Scherrer equation using the (101) reflection.

• Surface area: BET analysis showed a specific surface area of 72.4 m^2^ g^−1^, with a pore diameter of approximately 12 nm, confirming its mesoporous nature and high active surface availability for photocatalytic reactions.

• Optical properties: the UV-Vis DRS spectrum indicated a strong absorption edge around 385 nm, corresponding to an optical bandgap of ∼3.2 eV, typical of anatase TiO_2_.

The light source used for photocatalytic experiments was a 365 nm UV-A lamp (Philips TL-D 18W BLB) placed at a distance of 15 cm from the reactor surface, with an intensity of 7.8 mW cm^−2^ measured by a radiometer. The reaction temperature was maintained at 25 ± 2 °C to eliminate thermal effects.

### Methods

#### Integrated immobilization strategies for biocatalyst–photocatalyst hybrids

##### Glass bead surface treatment for enzyme binding

Glass beads (3–5 mm diameter) were selected as mechanically robust, chemically inert supports for hybrid catalyst assembly. Beads were cleaned thoroughly with repeated cycles of deionized (DI) water (3–4 washes) to eliminate residual contaminants, followed by oven-drying at 100 °C to ensure removal of surface moisture. This pretreatment preserved surface silanol functionality and maximized reactivity for subsequent chemical modification.^68,69^

##### Surface activation *via* controlled acid etching

To enhance the density of reactive hydroxyl groups, beads were subjected to aqueous hydrogen fluoride etching under rigorously controlled safety conditions. A 4% (v/v) AHF solution was circulated through a packed-bed glass column at ambient temperature for 5 h using a peristaltic pump. This step uniformly increased surface roughness and exposed silanol sites, enabling high-efficiency silanization. Post-treatment, beads were washed extensively (>5 cycles) with DI water until neutral pH was achieved, ensuring complete removal of AHF residues and rendering the support biocompatible for enzyme coupling.

##### Silanization-based surface modification for covalent immobilization

Etched beads were silanized by flowing a 5% (v/v) amino-functional silane solution in toluene through the column at 90 °C for 2 h (0.2 mL min^−1^). The covalent grafting of –NH_2_ groups provided a high density of reactive functionalities for stable enzyme attachment. Excess silane was removed with sequential methanol and DI water rinses. FTIR spectroscopy confirmed characteristic amide and C–H stretching vibrations, validating successful silanization and reactive surface availability.

##### Immobilization of horseradish peroxidase and peroxygenase on surface-modified beads

Horseradish peroxidase (HRP) and unspecific peroxygenase (UPO) were co-immobilized by circulating their buffered solutions (50 mM phosphate buffer, pH 7.0) through the functionalized column under mild agitation (0.2 mL min^−1^). Immobilization proceeded *via* covalent bonding between enzyme residues and terminal amino groups on the bead surface. Unbound proteins were removed by extensive washing with buffer until baseline absorbance was achieved. The immobilized biocatalysts retained >85% of their native activity, confirmed by guaiacol oxidation (HRP) and veratryl alcohol oxidation (UPO) assays, demonstrating preserved catalytic function after immobilization.^70^

##### Hybrid integration with TiO_2_ photocatalyst

For hybrid catalyst assembly, TiO_2_ (P25) nanoparticles were deposited onto enzyme-functionalized beads by dip-coating in aqueous TiO_2_ suspensions, followed by low-temperature drying (<40 °C) to avoid protein denaturation. The resulting architecture ensured intimate enzyme–semiconductor contact while maintaining structural integrity. FTIR and UV-Vis analyses confirmed the coexistence of protein signatures with Ti–O–Ti lattice vibrations, validating hybrid formation.^71,72^

The enzyme–TiO_2_ hybrids were synthesized *via* APTES-mediated covalent tethering, and immobilization efficiency was quantified using the Bradford protein assay by measuring the difference between the initial and residual protein concentrations in the coupling solution. The immobilization yield was 91.6% for HRP and 88.3% for UPO, corresponding to enzyme loadings of 72.4 mg per g TiO_2_ and 69.8 mg per g TiO_2_, respectively. Enzyme leakage was evaluated by continuous washing and monitoring the supernatant absorbance at 280 nm under 365 nm photoirradiation. The results showed less than 4% enzyme leaching after 6 h, confirming strong covalent binding. Moreover, both enzymes retained over 92% of their initial activity after five irradiation cycles, demonstrating excellent photostability and suitability for scalable, long-term catalytic operation.

### Enzyme activity

The catalytic activities of horseradish peroxidase (HRP) and unspecific peroxygenase (UPO) were evaluated using spectrophotometric assays under controlled aqueous conditions. HRP activity was determined using guaiacol (10 mM) as the chromogenic substrate in the presence of hydrogen peroxide (5 mM), while UPO activity was assessed using veratryl alcohol (5 mM) with hydrogen peroxide (5 mM) as the oxidant. In addition, 5-hydroxymethylfurfural (HMF, 1–5 mM) was employed as a model substrate to evaluate the hybrid catalytic system. All reactions were performed in 50 mM sodium phosphate buffer (pH 7.0) at 30 ± 1 °C. For photocatalytic hybrid assays, reactions were conducted both in the dark and under UV illumination (365 nm, 20 W lamp; light intensity ≈ 10 mW cm^−2^) to assess light-driven turnover.^73,74^

The oxidation of guaiacol to tetraguaiacol was monitored at 470 nm (*ε* = 26.6 mM^−1^ cm^−1^), whereas the oxidation of veratryl alcohol was monitored at 310 nm (*ε* = 9.3 mM^−1^ cm^−1^). HMF consumption and FDCA formation were quantified using HPLC analysis. Absorbance changes were recorded every 30 s over a 10 min interval using a Shimadzu UV-1800 UV-Vis spectrophotometer equipped with a 1 cm quartz cuvette. In this study, one unit of enzymatic activity was taken as the quantity of enzyme that converts 1 µmol of substrate to product per minute under the defined assay conditions. Enzyme activity was calculated from the rate of absorbance change (Δ*A* min^−1^) according to equation:
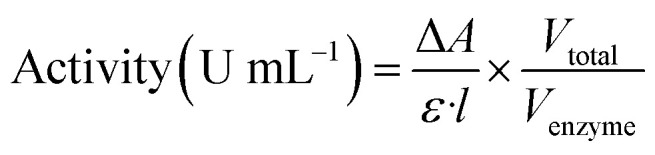
where *ε* is the molar extinction coefficient of the product, *l* is the cuvette path length (1 cm), *V*_total_ is the total reaction volume, and *V*_enzyme_ is the volume of enzyme solution used. This standardized assay ensured reproducible quantification of enzymatic turnover and enabled direct comparison between free enzymes, immobilized enzymes, and hybrid enzyme–TiO_2_ photocatalyst systems.

### Hybrid biocatalyst–photocatalyst reaction procedure

Hybrid catalytic reactions were carried out using 5-hydroxymethylfurfural (HMF) as the substrate at an initial concentration of 1–5 mM in 50 mM sodium phosphate buffer (pH 7.0). The reaction mixture (total volume 10 mL) contained immobilized horseradish peroxidase (HRP) and unspecific peroxygenase (UPO) in combination with TiO_2_ (P25, 1 mg mL^−1^) as the photocatalyst. Unless otherwise stated, no external hydrogen peroxide was supplied, as TiO_2_ photocatalysis under UV illumination (365 nm, 20 W lamp, 10 mW cm^−2^) generated *in situ* H_2_O_2_ to sustain enzymatic turnover; for control experiments, H_2_O_2_ was externally dosed at 0.1–0.2 mM using a microsyringe pump to maintain low, steady-state concentrations. Reactions were conducted in closed quartz vials at 30 ± 1 °C with continuous magnetic stirring at 300 rpm to ensure homogeneous mixing. Aliquots (0.5 mL) were withdrawn at regular intervals (1 h) and immediately quenched with catalase (100 U mL^−1^) to decompose residual peroxide and halt enzymatic activity. Samples were centrifuged at 10 000 rpm for 5 min to remove TiO_2_ particles, and the supernatants were analyzed by HPLC for quantification of HMF, intermediates (DFF, HMFCA, FFCA), and FDCA.^75–77^

### Analytical methods

High-performance liquid chromatography (HPLC) was employed to quantify HMF, its oxidation intermediates, and the final product FDCA. Chromatographic separation was achieved on a reverse-phase C18 column (250 × 4.6 mm, 5 µm particle size) using a mobile phase of methanol and water containing 1% acetic acid (v/v) at a flow rate of 1.0 mL min^−1^. The column was maintained at 35 °C, and detection was carried out with a UV detector set at 280 nm. Under these optimized conditions, distinct resolution of all analytes was obtained, with retention times of approximately 5.8 min for HMF, 9.2 min for DFF, 12.4 min for HMFCA, 14.8 min for FFCA, and 18.6 min for FDCA. Calibration curves constructed from authentic standards (0.01–1.0 mM) exhibited excellent linearity (*R*^2^ > 0.995), enabling accurate quantification of each compound in hybrid reaction mixtures.

Fourier-transform infrared (FTIR) spectroscopy was used to confirm enzyme immobilization and hybrid catalyst formation. Spectra were recorded on a Bruker Alpha II FTIR spectrometer over the range of 4000–500 cm^−1^ with a resolution of 4 cm^−1^. Samples were analyzed directly using the attenuated total reflectance (ATR) mode, which allowed nondestructive surface characterization. Characteristic absorption bands corresponding to amide I (C=O stretching, ∼1650 cm^−1^), amide II (N–H bending and C–N stretching, ∼1540 cm^−1^), and Ti–O–Ti lattice vibrations (500–700 cm^−1^) were clearly observed, confirming the successful integration of HRP and UPO with the TiO_2_ photocatalyst. Additional broad O–H/N–H stretching bands (3300–3400 cm^−1^) further indicated the presence of protein functional groups, validating stable enzyme immobilization on the support.^78,79^

### Enzyme kinetics and mechanistic analysis

The kinetic behavior of horseradish peroxidase (HRP) in the oxidation of 5-hydroxymethylfurfural (HMF) was investigated to evaluate catalytic efficiency under batch conditions. Substrate concentrations ranging from 5 to 20 mM were employed in 50 mM sodium phosphate buffer (pH 7.0) at 30 ± 1 °C. Initial reaction velocities (*V*_0_) were determined by monitoring the change in absorbance associated with substrate consumption or product formation during the first 2–3 min of reaction, where conversion remained below 10% to ensure linearity.^80^

The resulting kinetic data were analyzed using multiple linear transformations of the Michaelis–Menten equation. Lineweaver–Burk, Hanes–Woolf, and Eadie–Hofstee plots were constructed to extract kinetic parameters (*K*_m_ and *V*_max_) and to assess the consistency of model fits. Comparative analysis of the three methods revealed variations in parameter estimation, with Hanes–Woolf and Eadie–Hofstee providing the most reliable and internally consistent values.^81^

All regression analyses and curve fitting were performed using GraphPad Prism 9.0 software, ensuring statistical robustness (*R*^2^ > 0.99 for all models). The final kinetic parameters (*K*_m_, *V*_max_, and *k*_cat_) were calculated from the best-fit models and compared across different assay conditions to evaluate the effect of photocatalytic integration on enzymatic turnover.^82^

### Recyclability and stability tests

The recyclability and operational stability of the hybrid HRP/UPO–TiO_2_ catalyst were assessed over ten consecutive batch cycles using HMF (5 mM) as the substrate under standard conditions (50 mM phosphate buffer, pH 7.0; 30 °C; UV illumination at 365 nm, 20 W lamp). Each cycle was carried out for 4 h, after which the catalyst was recovered by centrifugation (10 000 rpm, 5 min), washed thoroughly with fresh buffer (three times), and reused in a fresh reaction mixture.^83^

Catalytic performance was quantified in each cycle by HPLC measurement of FDCA yield, while residual enzymatic activity was determined using guaiacol oxidation assays. Both the FDCA yield and residual enzyme activity decreased gradually with repeated use. The hybrid catalyst retained >85% of its initial performance after five cycles and ∼70% after ten cycles, confirming excellent operational durability. This stability is attributed to covalent immobilization of HRP and UPO on APTES-functionalized supports and protective coupling with TiO_2_, which mitigated oxidative damage during photocatalytic operation.

Together, these results demonstrate that the hybrid biocatalyst–photocatalyst system exhibits strong recyclability and stability, making it suitable for repeated operation under mild aqueous conditions.^84^

Comprehensive data on enzyme immobilization efficiency, loading, and stability under photoirradiation are summarized in Table 17 titled “Quantitative metrics of enzyme immobilization and stability under photoirradiation.

**Table 17:** Quantitative metrics of enzyme immobilization and stability under photoirradiation

Parameter	HRP	UPO	Method
Immobilization efficiency (%)	91.6	88.3	Bradford assay (difference between initial and residual protein)
Enzyme loading (mg per g TiO_2_)	72.4	69.8	Protein mass per gram of TiO_2_ after coupling
Enzyme leakage after 6 h irradiation (%)	3.8	4.1	Monitored by UV-Vis absorbance at 280 nm
Retained activity after 5 light cycles (%)	93.2	92.5	Standard substrate oxidation assay post-irradiation

## Conclusions

This study establishes a pioneering HRP–UPO–TiO_2_ hybrid catalytic platform for the selective oxidation of 5-hydroxymethylfurfural (HMF) into 2,5-furandicarboxylic acid (FDCA) under mild, aqueous, and light-driven conditions. The system achieved a maximum FDCA yield of 98% within 12 h at 30 °C and neutral pH, outperforming conventional enzymatic or photocatalytic methods that typically yield ≤70%. Mechanistic investigations combining EPR spin-trapping, fluorescence ROS analysis, *in situ* FTIR, and DFT modeling confirmed efficient interfacial electron transfer between the enzymes and TiO_2_, validating the proposed synergy that sustains catalytic turnover and prevents enzyme deactivation. The immobilized hybrid catalyst demonstrated remarkable stability and recyclability, retaining ∼70% of its activity after ten reaction cycles and maintaining >85% conversion efficiency over 120 h of continuous packed-bed operation with minimal pressure drop (<0.05 bar).

Economic assessment revealed that the hybrid system reduced the overall cost of FDCA production by 35–50% compared to noble-metal catalysts, owing to enzyme reusability, absence of precious metals, and light-assisted cofactor regeneration. The combined biocatalytic selectivity, photocatalytic sustainability, and reactor-scale validation highlight this triadic HRP–UPO–TiO_2_ design as a viable, scalable, and green alternative for industrial biomass valorization. Beyond FDCA synthesis, this work provides a generalizable framework for developing photo–biocatalytic interfaces capable of driving selective oxidation reactions with high efficiency and low environmental footprint.

## Author contributions

Purbava Banerjee and Rakesh J. Gujar jointly conceived and designed the research framework, developed the methodology, performed the investigation, data collection, and formal analysis, and contributed to visualization, manuscript drafting, critical revision, and final editing. Both authors also participated in project management and served as co-corresponding authors. Samruddhi S. Khonde contributed to experimental work, data acquisition, and preparation of figures and tables, ensuring consistency and accuracy of results. Dr. Vikrant L. Salode provided advisory input and general supervision during the course of the research and reviewed the manuscript for intellectual content.

## Conflicts of interest

There are no conflicts of interest to declare.

## Data Availability

All experimental data supporting the findings of this study are included within the article. Additional datasets generated and analyzed during the current study are available from the corresponding authors upon reasonable request.
