# Correction: First hybrid enzyme–photocatalyst synergy for sustainable biomass conversion

**DOI:** 10.1039/d6ra90003h

**Published:** 2026-01-26

**Authors:** Rakesh J. Gujar, Samruddhi S. Khonde, Purbava Banerjee, Vikrant L. Salode

**Affiliations:** a Independent researcher India rakeshgujar0898@gmail.com; b P. R. Patil Institute of Pharmacy Talegaon (S.P.) Maharashtra 442201 India; c Department of Bioprocess Technology, Institute of Chemical Technology (ICT) Mumbai Maharashtra 400019 India

## Abstract

Correction for ‘First hybrid enzyme–photocatalyst synergy for sustainable biomass conversion’ by Rakesh J. Gujar *et al.*, *RSC Adv.*, 2025, **15**, 50714–50733, https://doi.org/10.1039/d5ra07500a.

The authors regret that the name of one of the authors (‘Samruddhi S. Khonde’) was inaccurately shown in the original article. The corrected author list is as shown above.

The authors regret that some of the affiliations were incorrectly shown in the original manuscript. The corrected list of affiliations is as shown here.

The authors regret that the order of authors in the author list was shown incorrectly in the original article. The corrected author list is as shown above.

The Royal Society of Chemistry apologises for these errors and any consequent inconvenience to authors and readers.

